# Influence of N_2_ on Formation Conditions and Guest Distribution of Mixed CO_2_ + CH_4_ Gas Hydrates

**DOI:** 10.3390/molecules23123336

**Published:** 2018-12-15

**Authors:** Vladimir R. Belosludov, Yulia Yu. Bozhko, Oleg S. Subbotin, Rodion V. Belosludov, Ravil K. Zhdanov, Kirill V. Gets, Yoshiyuki Kawazoe

**Affiliations:** 1 Nikolaev Institute of Inorganic Chemistry, SB RAS, Novosibirsk 630090, Russia; bel@niic.nsc.ru (V.R.B.); subbot@niic.nsc.ru (O.S.S.); rav@niic.nsc.ru (R.K.Z.); gets@niic.nsc.ru (K.V.G.); 2 Department of Physics, Novosibirsk State University, Pirogova Str. 2, Novosibirsk 630090, Russia; 3 Institute for Materials Research, Tohoku University, Sendai 980-8577, Japan; rodion@imr.tohoku.ac.jp; 4 New Industry Hatchery Center, Tohoku University, Sendai 980-8579, Japan; kawazoe@e-workshop.co.jp; 5 SRM institute of Science and Technology, Kattankulathur 603203, India

**Keywords:** gas separation, lattice dynamic, mixed gas hydrates, greenhouse gases, computer modeling

## Abstract

In this contribution, a method based on a solid solution theory of clathrate hydrate for multiple cage occupancy, host lattice relaxation, and guest-guest interactions is presented to estimate hydrate formation conditions of binary and ternary gas mixtures. We performed molecular modeling of the structure, guest distribution, and hydrate formation conditions for the CO_2_ + CH_4_ and CO_2_ + CH_4_ + N_2_ gas hydrates. In all considered systems with and without N_2_, at high and medium content of CO_2_ in the gas phase, we found that CO_2_ was more favorable in occupying clathrate hydrate cavities than CH_4_ or N_2_. The addition of N_2_ to the gas phase increased the ratio concentration of CO_2_ in comparison with the concentration of CH_4_ in clathrate hydrates and made gas replacement more effective. The mole fraction of CO_2_ in the CO_2_ + CH_4_ + N_2_ gas hydrate rapidly increased with the growth of its content in the gas phase, and the formation pressure of the CO_2_ + CH_4_ + N_2_ gas hydrate rose in comparison to the formation pressure of the CO_2_ + CH_4_ gas hydrate. The obtained results agreed with the known experimental data for simple CH_4_ and CO_2_ gas hydrates and the mixed CO_2_ + CH_4_ gas hydrate.

## 1. Introduction

Due to concerns of an increasing global warming effect, the capture from industrial flue gas and long-term storage of carbon dioxide are among the most important challenges facing the world scientific community today. 

Various strategies [1] to sequester carbon dioxide have been proposed, but a technology for large-scale and safely stored CO_2_ has not been completely developed.

Currently, the leading approach to this problem involves the injection of CO_2_ into depleted deep underground natural gas reservoirs [2]. In seismically active zones such as Japan, the use of this method of CO_2_ storage may lead to gas leakage due to geological perturbations such as earthquakes or fractures. Another approach offered by Ohgaki and Inoue [3] is the sequestration of CO_2_ as solid hydrates through the formation of CO_2_ clathrate hydrate. Recently, the injection of CO_2_ into porous sediments at a depth of several hundred meters below the deep ocean floor has been proposed as an alternative long-term sequestration option that would be resistant to geophysical perturbations [4]. Such deposition of CO_2_ prevents the transport of the CO_2_ back to the surface due to the formation of a CO_2_ clathrate hydrate capping layer that reduces the migration. The authors estimate this storage strategy could remain intact for millions of years. It has also been shown that CO_2_ hydrates have anomalously low dissociation rates at atmospheric pressure. This self-preservation effect takes place in the temperature range 245–271 K, which could be significant in practice for the CO_2_ storage in the form of clathrate hydrates [5].

Clathrate hydrates are nonstoichiometric inclusion compounds consisting of water (host) molecules forming a crystalline framework in which cavities (cages) guest molecules can be included. In nature, three types of gas hydrates are common: Hydrates of cubic structure (CS)-I, cubic structure CS-II, and hexagonal structure (HS)-III [6]. The hydrate structure is determined primarily by the size of guest molecules. Thus, large guest molecules such as propane and isobutane as well as small guests, in particular oxygen, nitrogen, and hydrogen, form the cubic structure CS-II. Guest molecules of intermediate size such as methane, xenon, and carbon dioxide form hydrates of the cubic structure CS-I. The presence of two types of guest molecules is necessary to form the hexagonal structure HS-III: A very large molecule (e.g., 2,2-dimethylbutane) and a small molecule (e.g., methane). The hydrate structures differ by size and number of cavities in their unit cells. 

Large amounts of natural gas hydrates are composed mainly of methane in the form of solid hydrates stored on continental margins and in permafrost regions [6]. Technologies involving the simultaneous production of raw hydrocarbon and greenhouse-gas sequestration is promising. Carbon dioxide sequestration in deep-sea sediments or permafrost regions can be performed simultaneously with natural gas recovery by swapping hydrocarbon molecules in hydrate cages for carbon dioxide molecules, thus providing a mechanism of hydrocarbons production and greenhouse gas sequestration [7,8,9]. The replacement of CH_4_ hydrates by CO_2_ hydrates has been studied [7] for recovering CH_4_ gas. When a mixture of water with gas or liquid CO_2_ itself is put under certain pressure, a solid CO_2_ hydrate can be formed at much milder *P-T* conditions than a CH_4_ hydrate can [10]. Thus, the swapping process between two gaseous guests is considered to be a promising approach to long-term storage of CO_2_. When the CH_4_ hydrate is put under a certain pressure [8,9] of a CO_2_/N_2_ gas mixture, a decomposition of CH_4_ hydrates and a solid mixed hydrate containing CO_2_ can appear with recovered CH_4_ gas. The direct use of a CO_2_ + N_2_ gas mixture (20 mol% CO_2_ and 80 mol% N_2_ to reproduce flue gas from a power plant) instead of pure CO_2_ greatly enhances the overall CH_4_ recovery rate in complex marine systems and reduces the costs [11] of CO_2_ separation from flue gas. A great number of experimental and theoretical studies concerning the stability and composition of gas hydrates formed from gas mixtures has been published in the last decade [6,12,13,14,15,16,17,18,19,20,21,22]. In particular, a comparison of numerous experimental data on phase equilibria in a water–methane–carbon dioxide and a water–nitrogen–carbon dioxide systems were presented in papers [23,24,25,26,27,28]. The stability of CO_2_/N_2_ or CO_2_/CH_4_ mixed hydrates were studied in various conditions, and it was shown that the three-phase hydrate–water–vapor equilibrium curves were shifted to higher pressures at all considered temperatures with decreases [25,26,27,28] in CO_2_ concentration in the vapor phase. The statistical thermodynamic theory of van der Waals and Platteeuw [29] was used for modeling the hydrate phase containing nitrogen [23,24,25]. The main assumptions were made in the original van der Waals–Platteeuw model: It stated that each cavity can contain at most one gas molecule. However, Kuhs and coworkers [30,31] found the first direct evidence for multiple occupancy of the cages in nitrogen hydrate. These results have been confirmed by molecular dynamics simulations of CS-II nitrogen hydrate with varying cage occupancies and at different conditions [32,33]. For the more correct prediction of hydrate phase equilibria, it is necessary to consider the possibility of multiple occupancy of the cages in the gas hydrate containing nitrogen. 

The aim of this paper is the investigation of the possibility of recovering methane from methane hydrates using either CO_2_ or a CO_2_/N_2_ gas mixture. The hydrate phase was treated with the solid solution theory of clathrate hydrate for multiple cage occupancy, host lattice relaxation, and guest-guest interactions [34,35,36,37,38]. With this goal, we determined the dependencies of the compositions of the gas hydrates formed from methane + carbon dioxide, nitrogen + carbon dioxide binary gas mixtures, and methane + carbon dioxide + nitrogen ternary gas mixtures, as well as of the formation conditions of these hydrates in dependence on temperature and pressure for different compositions of the gas phase.

## 2. Methods

### 2.1. Implemented Theoretical Models

In order to accurately estimate the thermodynamic properties of clathrate hydrates, we developed an approach based on the solid solution theory of clathrate hydrate for multiple cage occupancy, host lattice relaxation, and guest-guest interactions [34,35,36,37,38]. The method is based on only one of several assumptions of the original van der Waals–Platteeuw theory [29]: The free energy of clathrate hydrate does not depend on the arrangement of guest molecules in cavities at fixed values of filling degrees for each definite type of cavity. In this approach, the lattice dynamics method that takes quantum effects into account is used and the crystalline host lattice is considered to be nonrigid, is able to change volume depending on the type of guest molecules, and is permitted to describe first-order phase transitions.

The mathematical formalism of the present model for the general case and in the case of clathrate hydrates with two types of cavities and one type of guest was described in our previous studies [34,35]. In contrast with our previous work, in the present work we formulated our approach for the hydrate having two types of cavities, large (L) and small (S), and with the possibility of single occupancy of small and large cavities by a, b, and c type guests; single occupancy of large cavities by a,b type guests; and multiple occupancy of large cavities by c type guests.

In the mentioned approximation, free energy of the clathrate hydrates could be presented as

(1) $$F=F_{1}\left(V,T,\left\{y\right\}\right)+k_{Β}T\sum_{t,l,i_{l}}N_{t}\left[\left(1-\sum_{t,l,i_{l}}y_{t}^{i_{l}l}\right)\ln\left(1-\sum_{t,l,i_{l}}y_{t}^{i_{l}l}\right)+\frac{y_{t}^{i_{l}l}}{i_{l}!}\ln y_{t}^{i_{l}l}\right],$$

where:

(a) For binary clathrate hydrates with cavities of two types including two types of guest molecules and the possibility of single occupancy for type a guests and single occupancy for type b guests of both the small and large cavities, $i_{l}=1$, l=a,b*,* t=S,L; $F_{1}$ is the part of free energy at a given degree of filling of the guest molecules in the S and L cavities; $\left\{y\right\}=\left\{y_{S}^{a},y_{L}^{a},y_{S}^{b},y_{L}^{b}\right\}$, $y_{t}^{l}=N_{t}^{l}/N_{t}$ are filling degrees for cavities of the tth type (t=S,L) by guest molecules of the lth type (l=a,b); $N_{t}$ is the number of cavities of the tth type; and $N_{t}^{l}$ is the number of guest molecules of the lth type contained in cavities of the tth type.
(b) For ternary clathrate hydrates with cavities of two types including three types of guest molecules and the possibility of single occupancy for type a guests and single occupancy for type b guests of both the small and large cavities, as well as the possibility of single occupancy for type c guests of the small and multiple occupancy large cavities, l=a,b,c*,* $i_{a}=1$*,* $i_{b}=1$*,* $i_{c}=1,2$*,* t=S,L; $F_{1}$ is the part of free energy at a given degree of filling of the guest molecules in the S and L cavities; $\left\{y\right\}=\left\{y_{S}^{a},y_{L}^{a},y_{S}^{b},y_{L}^{b},y_{S}^{c},y_{L}^{c},y_{L}^{2c}\right\}$, $y_{t}^{i_{l}l}=N_{t}^{i_{l}l}/N_{t}$ is the filling degree for a cavity of the tth type (t=S,L) by guest molecules of the lth type (l=a,b,c); $N_{t}$ is the number of cavities of the tth type; $N_{t}^{i_{l}l}$ is the number of guest molecules of the lth type contained in cavities of the tth type.

In our cases, the binary clathrate hydrates were $i_{l}=1$, $l=CO_{2},CH_{4}$, t=S,L, and the ternary clathrate hydrates were $i_{CO_{2}}=1$, $i_{CH_{4}}=1$, $i_{N_{2}}=1,2$, $l=CO_{2},CH_{4},N_{2}$, t=S,L. In the models, it was considered that the molecules of *CO*_2_, *CH_4_*, and *N*_2_ could single occupy both the small and large cavities, whereas molecules of *N*_2_ could also double occupy both the large cavities.

For a given arrangement {y} of the guest molecules in the cavities the free energy $F_{1}\left(V,T,\left\{y\right\}\right)$ of the crystal could be calculated within the framework of a lattice dynamics approach as

(2) $$F_{1}\left(V,T,\left\{y\right\}\right)=U+F_{\mathrm{vib}},$$

where U is the potential energy, and $F_{vib}$ is the vibrational contribution

(3) $$F_{\mathrm{vib}}=\frac{1}{2}\sum_{j\vec{q}}\hbar \omega_{j}\left(\vec{q}\right)+k_{Β}T\sum_{j\vec{q}}\ln\left(1-\exp\left(-\hbar \omega_{j}\left(\vec{q}\right)/k_{Β}T\right)\right),$$

where $\omega_{j}\left(\vec{q}\right)$ is the jth eigenfrequency of crystal vibration, and $\vec{q}$ is the wave vector. Free energy was computed for several values of volume, and it had a minimum corresponding to the equilibrium structure at zero pressure.

The equation of state was found by numerical differentiation of the free energy with respect to volume:

(4) $$P\left(V,T\right)=-{\left(\frac{\partial F\left(V,T,\left\{y\right\}\right)}{\partial V}\right)}_{0}.$$

Then we found the chemical potentials $\mu_{t}^{i_{l}l}$ of guest molecules in the hydrate by numerical differentiation of the free energy with respect to the number of guest molecules:

(5) $$\mu_{t}^{i_{l}l}\left(P,T,\left\{y\right\}\right)={\left(\frac{\partial F\left(V,T,\left\{y\right\}\right)}{\partial N\mu_{t}^{i_{l}l}}\right)}_{0}={µ}_{t}^{*li_{l}}+k_{B}\mathrm{Tln}\frac{y_{t}^{i_{l}l}}{i_{l}!\left(1-\sum_{t,l,i}y_{t}^{i_{l}l}\right)},$$

(6) $${µ}_{t}^{*li_{l}}={\left(\frac{\partial F_{1}\left(V\left(P\right),T,\left\{y\right\}\right)}{\partial N_{t}^{i_{l}l}}\right)}_{0}.$$

If the free energy F is known, then the Gibbs free energy is

(7) $$\begin{array}{c} \Phi \left(P,T,\left\{y\right\}\right)=N_{Q}\mu_{Q}\left(P,T,\left\{y\right\}\right)+\sum_{t,l,i}N_{t}^{i_{l}l}\mu_{t}^{i_{l}l}\left(P,T,\left\{y\right\}\right)=F\left(V\left(P\right),T,\left\{y\right\}\right)+\mathrm{PV}\left(P\right) \end{array}.$$

As an expression in terms of the chemical potentials of the host and guest molecules, it can be found that

(8) $$\mu_{Q}\left(P,T,\left\{y\right\}\right)={µ}_{Q}^{*}\left(\left\{y\right\}\right)+k_{B}T\sum_{t,l,i_{l}}\nu_{t}\left(1-\sum_{t,l,i_{l}}y_{t}^{i_{l}l}\right),$$

(9) $${µ}_{Q}^{*}\left(P,T,\left\{y\right\}\right)=\frac{1}{N_{Q}}F_{1}\left(V\left(P\right),T,\left\{y\right\}\right)+\frac{1}{N_{Q}}\mathrm{PV}\left(P\right)-\sum_{t,l,i_{l}}\nu_{t}y_{t}^{i_{l}l}{µ}_{t}^{*li_{l}}\left(P,T,\left\{y\right\}\right),$$

where $\nu_{t}=\frac{N_{t}}{N_{Q}}$**,** and $N_{Q}$ is the number of water molecules.

The *P*-*T* line of monovariant equilibrium of different hydrates and ices could be found from the equality condition of the chemical potentials of water molecules in hydrates and in ice or in the liquid phase:

(10) $$\mu_{Q}\left(P,T,\left\{y\right\}\right)=\mu_{Q}^{\mathrm{ice}}\left(P,T\right);\mu_{Q}\left(P,T,\left\{y\right\}\right)=\mu_{{\mathrm{wA}}_{g}}\left(P,T\right).$$

Analogously, the equality of the chemical potentials of guest molecules in the hydrate and gas phases could be written as

(11) $$\mu_{t}^{i_{l}l}\left(P,T,\left\{y\right\}\right)=\mu_{i_{l}l}^{g}\left(P,T\right).$$

The chemical potential of guest molecules in the gas phase were calculated using the following equations for a non-ideal gas mixture with a Lennard–Jones interaction between molecules [20]:

(12) $$\mu_{i}^{g}\left(V,T\right)=k_{B}T\ln\left[\frac{N_{i}^{g}}{V}{\left(\frac{2\pi \hbar^{2}}{m_{i}T}\right)}^{3/2}\right]-\frac{\partial }{\partial N_{i}^{g}}\left({\mathrm{Nk}}_{B}T\ln\left(1-N\frac{\sum_{i,j}\varepsilon_{\mathrm{ij}}x_{i}^{g}x_{j}^{g}}{V}\right)-N^{2}\frac{\sum_{i,j}\sigma_{\mathrm{ij}}x_{i}^{g}x_{j}^{g}}{V}\right),$$

where $\varepsilon_{i}$ and $\sigma_{i}$ are Lennard–Jones parameters. Interaction parameters between molecules of different types are defined by the combination rules $\varepsilon_{ij}=\sqrt{\varepsilon_{i}\varepsilon_{j}}$ and $\sigma_{ij}=\frac{\sigma_{i}+\sigma_{j}}{2}$:$x_{i}=N_{i}^{g}/N^{g}$ is the mole fraction of the ith component in the gas mixture,$N^{g}$ is the number of guest molecules in the gas phase; $N_{i}^{g}$ is the number of guest molecules of the lth type in the gas phase; and $m_{i}$ is the molar mass of the ith component. The first term in Equation (12) corresponded to the chemical potential of the ideal gas, and the second two corrections appeared for real gases.

The chemical potential of liquid water $\mu_{{\mathrm{wA}}_{q}}$ was taken from the model proposed earlier [39] and was given by

(13) $$\mu_{{\mathrm{wA}}_{q}}\left(P,T\right)=\frac{T}{T_{0}}g_{w_{0}L_{\mathrm{pure}}}-\int_{T_{0}}^{T}\frac{dT^{\prime }}{T}h_{{\mathrm{wL}}_{\mathrm{pure}}}+\int_{P_{0}}^{P}v_{{\mathrm{wL}}_{\mathrm{pure}}}dP^{\prime },$$

where $T_{0}$ is the initial temperature, and $P_{0}$ is the initial pressure. The following constants were also used, as defined in Reference [39]: The Gibbs energy of formation $g_{w_{0}L_{\mathrm{pure}}}$, the molar enthalpy of water $h_{{\mathrm{wL}}_{\mathrm{pure}}}$, and the water volume $v_{{\mathrm{wL}}_{\mathrm{pure}}}$, all constants that refer to pure water. Instead of using the empirical value of $g_{w_{0}L_{\mathrm{pure}}}$, we calculated this parameter directly by using the lattice dynamic method [38]. The constants were recalculated because those given in Reference [39] were related to standard conditions. We calculated the Gibbs energy and enthalpy at *T* = 273.15 K (i.e., the temperature of the equilibrium between the ice and water phases, but with pressure remaining at 1 atmosphere). It was connected with the differences between experimental Gibbs energy and that estimated within the model’s interaction potential. 

This parameter could be evaluated from the chemical potential of water, $\mu_{{\mathrm{wA}}_{q}}$, which should be equal to the $\mu_{0}^{ice}$ of hexagonal ice calculated at the ice $I_{h}$ melting point at standard pressure and temperature. The degrees of cage filling were found from Equations (5) and (11):

(14) $$y_{t}^{i_{l}l}=\frac{\beta_{t}^{i_{l}l}}{1+\beta_{t}^{i_{l}l}};\beta_{t}^{i_{l}l}=\exp\left\{\frac{1}{\mathrm{kT}}\left[\mu_{l}^{\mathrm{gas}}-{µ}_{t}^{*li_{l}}\right]\right\}.$$

For determination of the hydrate composition as a function of the gas phase composition, the following relations for fractions of the filled large ($C_{L}^{i_{l}l}$) and small ($C_{S}^{i_{l}l}$) cages were used, and mole fractions in guest subsystems of l type guest molecules included in the hydrate phase ($x_{l}^{h}$) were used:

(15) $$C_{t}^{i_{l}l}=\frac{y_{t}^{i_{l}l}N_{t}}{\sum_{t,l,i_{l}}y_{t}^{i_{l}l}N_{t}};x_{l}^{h}=\sum_{i_{l},t}C_{t}^{i_{l}l}.$$

### 2.2. Simulations Details

The unit cells were chosen as the simulation cell of CS-I (46 water molecules forming 6 large and 2 small cages) and CS-II hydrates (136 water molecules forming 8 large and 16 small cages). Large cages as well as small ones could be filled by one carbon dioxide or methane molecule. The possibility of double filling of large or small cages by these guest molecules was not considered due to the comparatively large size of these molecules. For modeling of ice $I_{h}$, the simulation supercell containing 32 unit cells (i.e., 128 water molecules) was used. Coulomb interactions were calculated by the Ewald method. The protons were placed according to Bernal–Fowler rules [40], and the water molecules were oriented such that the total dipole moments of the simulation cells of ice and the hydrates were zero with a precision of better than 0.1% of the magnitude of the dipole moment of a single water molecule. The interaction of water–water molecules in hydrates and in ice were described by the modified SPC/E (Simple Point Charge/Extended) potential [41]:

(16) $$U_{\mathrm{ij}}\left(r\right)=4\varepsilon_{\mathrm{ij}}\left[{\left(\frac{\sigma_{\mathrm{ij}}}{r}\right)}^{12}-{\left(\frac{\sigma_{\mathrm{ij}}}{r}\right)}^{6}\right]+\frac{q_{i}q_{j}}{r},$$

where the Lennard–Jones parameters are $\sigma_{O}=3.1556$ Å, $\varepsilon_{O}=0.65063$ kJ/mole. The usage of other water–water interactions models could influence the absolute values of chemical potentials and slightly influence the ice–gas–hydrate equilibria. Although the chosen water–water interaction model was simple, it was in agreement with known experimental data and was earlier successively applied to consider properties of a number of other simple and mixed hydrates such as CH_4_, C_2_H_6_, Xe, CH_4_ + C_2_H_6_, and it allowed us to establish the correctness of our model [34].

Charges on hydrogen atoms were $q_{H}=+0.4238\left|e\right|$ and on oxygen atoms $q_{O}=-0.8476\left|e\right|$. This parameters selection allowed us to reach good agreement with the experimental data [34,35]. For description of the interactions of guest molecules between each other and with water molecules, the Lennard–Jones potential was used with the parameters σ=3.73 Å, ε=1.2305 kJ/mole for methane molecules [42], σ=4.00 Å, ε=1.5801 (1) kJ/mole for carbon dioxide molecules [43], and σ=3.6154 Å, ε=0.844 kJ/mole for nitrogen molecules [44].

## 3. Results and Discussion

### 3.1. Gas–Hydrate Phase Equilibria

**Figure 1:**
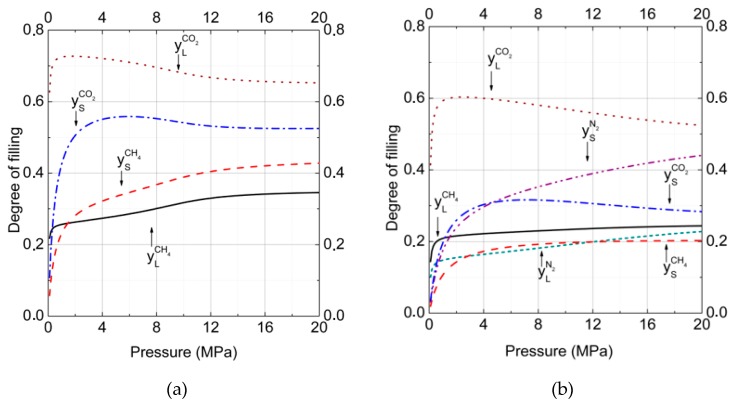
Degree of cage filling for large and small cavities in hydrate at equilibrium conditions at *T* = 277 K. Gas phase mole fractions were (**a**) a binary mixture containing carbon dioxide (50%) and methane (50%) and (**b**) a ternary mixture containing carbon dioxide (15%), methane (15%), and nitrogen (70%) ($y_{L}^{CH_{4}}$ = solid; $y_{S}^{CH_{4}}$ = dashed; $y_{L}^{CO_{2}}$ = dotted; $y_{S}^{CO_{2}}$ = dash-dotted; $y_{S}^{N_{2}}$ = dash-dot-dotted; and $y_{L}^{N_{2}}$ = short dashed lines). The total number of calculated points was equal to 200 with a pressure step of 0.1 MPa for each curve.

Gas–hydrate divariant equilibria are described by Equation (11). This equation represents the conditions of equality of chemical potentials of guest molecules in a hydrate with the gas phase of the same kind of molecules in dependence on pressure and temperature. The comparison of degrees of filing for binary (50% CH_4_ and 50% CO_2_ in the gas phase) and ternary (15% CH_4_ and 15% CO_2_ and 70% N_2_ in the gas phase) mixed hydrates at the temperature *T* = 277 K are presented in Figure 1a,b. One can see (Figure 1a) that occupation of both small and large cavities by carbon dioxide molecules was more preferable than by methane. The difference in the degrees of filling was the result of a slightly larger size of CO_2_ molecules and stronger interaction with water molecules. After the addition of nitrogen into the gas phase, the tendency was the same (Figure 1b), but in this case cavities’ occupation by N2 molecules could be concurred by CO_2_ and CH_4_ molecules. In spite of more than a two times higher concentration of nitrogen in the gas phase, methane and carbon dioxide molecules more rapidly occupied the large cavities. Thus, at the pressure 10 MPa, only 19% of large cavities were filled by N_2_, whereas 24% and 57% were filled by CH_4_ and CO_2_, respectively. A different situation was observed for small cavities filling. In this case, N_2_ molecules became preferable and could concur with larger molecules of CH_4_ and CO_2_. Therefore, at the same pressure, 10 MPa, the cages were filled by N_2_, CH_4_, and CO_2_ in the amounts of 37%, 19%, and 31%, respectively. A tendency of increasing of the cavities number occupied by nitrogen molecules with the pressure increasing was also observable. In both binary and ternary hydrates, one could see the noticeable growth of ratios $\frac{y_{L}^{CO_{2}}}{y_{L}^{CH_{4}}}$ and $\frac{y_{S}^{CO_{2}}}{y_{S}^{CH_{4}}}$ with the nitrogen addition. For binary the CO_2_ + CH_4_ hydrate, $\frac{y_{L}^{CO_{2}}}{y_{L}^{CH_{4}}}=2.09$, whereas for the ternary CO_2_ + CH_4_ + N_2_ hydrate, $\frac{y_{L}^{CO_{2}}}{y_{L}^{CH_{4}}}=2.38$. At the same time, for the binary CO_2_ + CH_4_ hydrate, $\frac{y_{S}^{CO_{2}}}{y_{S}^{CH_{4}}}=1.38$, and for the ternary CO_2_ + CH_4_ + N_2_hydrate $\frac{y_{S}^{CO_{2}}}{y_{S}^{CH_{4}}}=1.63$. This meant that with the addition of N_2_ to the gas phase, nitrogen molecules more readily displaced CH_4_ than CO_2_ molecules. This occurred because of higher guest–host (H_2_O) interaction energy and a slightly larger van der Waals radius of CO_2_ in comparison to CH_4_.

**Figure 2:**
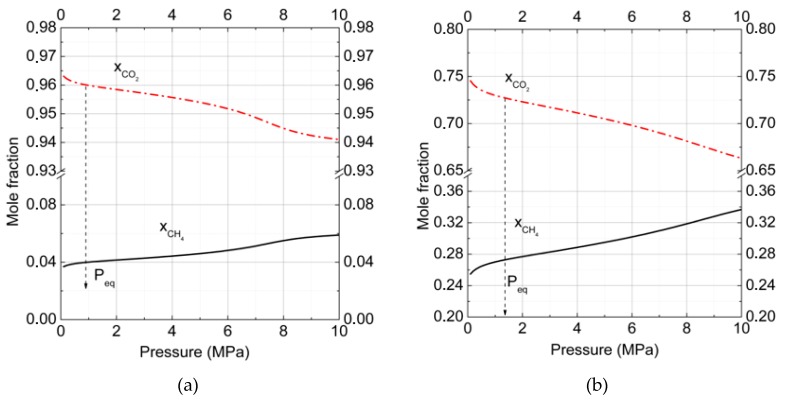
Mole fraction of carbon dioxide and methane in binary hydrates at *T* = 273 K. The gas phase consisted of (**a**) carbon dioxide (90%) and methane (10%) and (**b**) carbon dioxide (50%) and methane (50%) ($x_{CH_{4}}$= solid and $x_{CO_{2}}$ = dash-dotted lines). The total number of calculated points was equal to 100 with a pressure step of 0.1 MPa for each curve.

**Figure 3:**
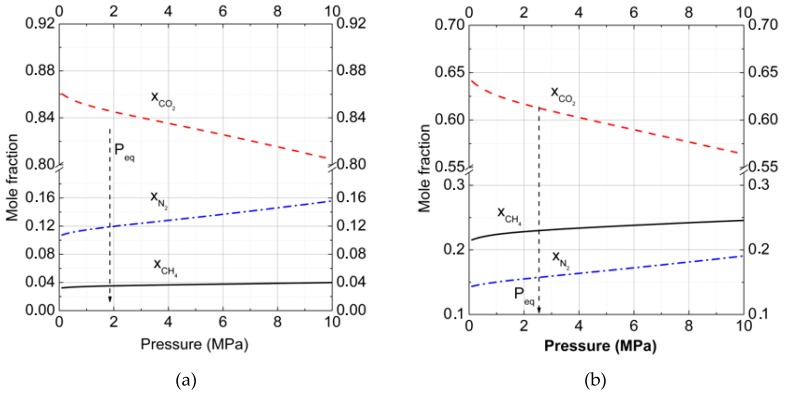
Mole fraction of carbon dioxide, methane, and nitrogen in ternary hydrate phases at *T* = 273 K. The gas phase consisted of (**a**) carbon dioxide (27%) and methane (3%) with nitrogen (70%), and (**b**) carbon dioxide (15%), methane (15%), and nitrogen (70%) ($x_{CH_{4}}$= solid; $x_{CO_{2}}$ = dashed; and $x_{N_{2}}$ = dash-dotted lines). The total number of calculated points was equal to 100 with a pressure step of 0.1 MPa for each curve.

Figure 2a,b show the change of CO_2_ and CH_4_ mole fractions in binary hydrates in dependence on pressure for two gas phase compositions at *T* = 273 K. Analogous data of mole fractions changed for CO_2_, CH_4_, and N_2_ in ternary hydrates at *T* = 273 K and are presented in Figure 3. The arrows show the equilibrium formation points for hydrates. The intriguing result was that with pressure increasing, the CO_2_ fraction in hydrate decreased while the CH_4_ fraction grew. Such behavior correlated with results for the filling of large and small cavities (Figure 1a).

Another interesting finding was that the CO_2_ fraction decrease rate in the hydrate phase was small for high CO_2_ concentrations in the gas phase (Figure 2a), and it increased with CO_2_ concentration decreases. At the temperature *T* = 273 K (Figure 2a,b), in the pressure interval 1 to 10 MPa for the gas mixture of 90% CO_2_ and 10% CH_4_, the change of $x_{CH_{4}}$ or $x_{CO_{2}}$ was about 0.022 whereas for the gas mixture of 50% CO_2_ + 50% CH_4_ it became about 0.082, almost four times larger.

After the addition of nitrogen to the binary carbon dioxide + methane mixture (Figure 3), the amount of these gases stored in the hydrate phase decreased, but not drastically. At the formation pressure (1.8 MPa at *T* = 273 K) for the ternary gas mixture of 27% CO_2_ + 3% CH_4_ + 70% N_2_, (Figure 3a), the relative fraction of CO_2_ in the hydrate was found to be 0.847 instead of 0.96 for the binary mixture 90% CO_2_ + 10% CH_4_. Methane with its low content in the gas phase (3%) had a fractional content of 0.035. In comparison to the hydrate phase formed from the ternary gas mixture 15% CO_2_ + 15% CH_4_ + 70% N_2_ (Figure 3b), the situation changed notably. At first, methane occupied a notable part of hydrate cavities and the mole fraction of methane reached 0.229 at the formation pressure. Nitrogen content in hydrate became 0.163, almost 40% higher than in the previous case. Methane and nitrogen could replace carbon dioxide in cavities, but CO_2_ molecules still occupied more than 60% of cavities (mole fraction is 0.614). The increase of relative gas fraction contents in hydrates with pressure was almost equal for different gas phase compositions (0.06 for 27% CO_2_ and 0.08 for 15% CO_2_ in gas mixtures).

In all considered systems with and without N_2_, at high and medium content of CO_2_ in the gas phase, we found that CO_2_ was more favorable in occupying clathrate hydrate cavities than CH_4_ or N_2_. Moreover, the addition of N_2_ to the gas phase increased the $\frac{x_{CO_{2}}}{x_{CH_{4}}}$ ratio. For mixtures of 50% CO_2_ + 50% CH_4_ and 15% CO_2_ + 15% CH_4_ + 70% N_2_, this ratio increased by 1.5%, and for mixtures of 90% CO_2_ + 10% CH_4_ and 27% CO_2_ + 3% CH_4_ + 70% N_2_, by about 1%. Therefore, the addition of N_2_ made gas replacement more effective.

### 3.2.Gas–Hydrate–Ice (Water)Phase Equilibria

We conducted a calculation of *P*-*T* diagrams for gas–hydrate–ice (water) phase equilibria (described by Equations (10) and (11), carried out earlier) for one-component hydrates of methane and reproduced the experimental data with good accuracy [45,46]. 

**Figure 4:**
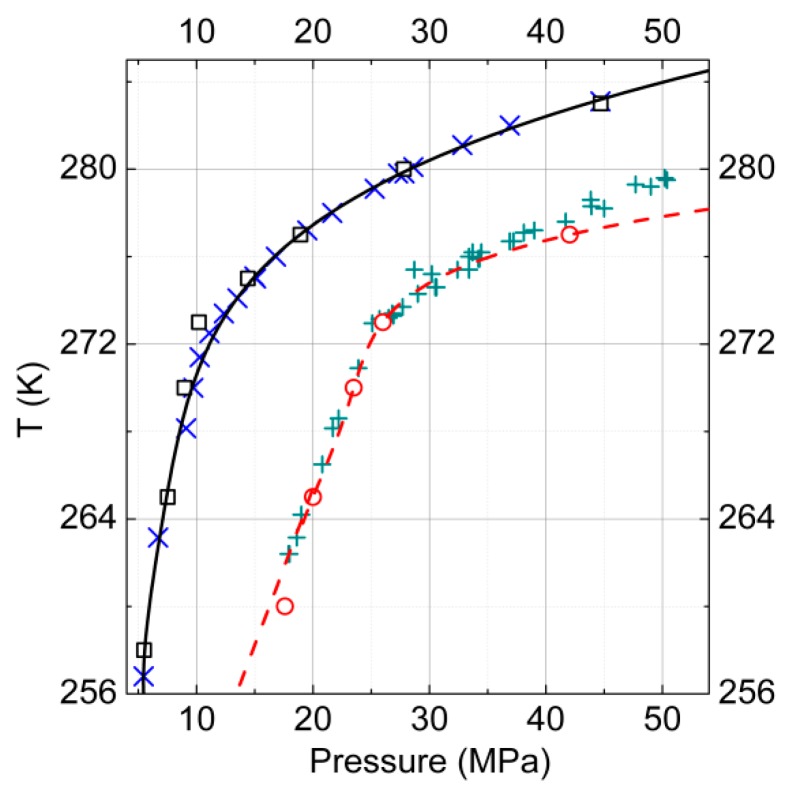
*P*-*T* diagram of gas–hydrate–ice (water) phase equilibria for one-component hydrates of carbon dioxide and methane. The results of the calculations for carbon dioxide are represented by the open squares, and the experimental data [6] by the skew crosses. For methane, the calculated data are shown by open circles and the experimental data [6] by crosses.

The modeling was performed within the molecular model framework described above: Here such calculations were conducted for carbon dioxide hydrates. The obtained lines of the phase equilibria also were in reasonable agreement with the experimental data. In Figure 4, the calculated curves of the ice–gas–hydrate phase equilibria are presented for the considered one-component hydrates of carbon dioxide and methane as well as the available experimental data [6] for comparison.

**Figure 5:**
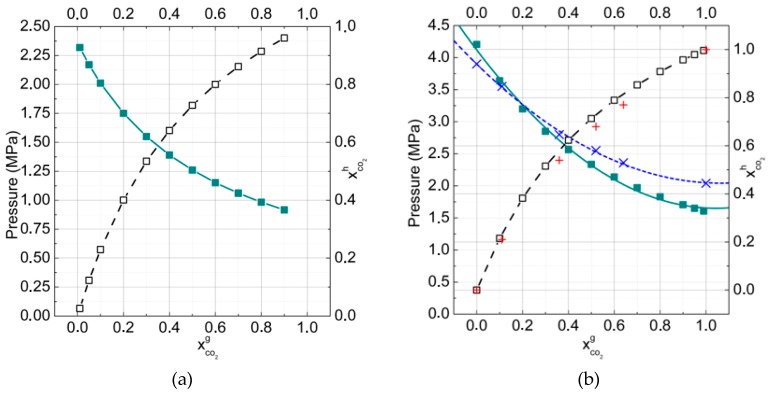
*P*-*x* diagram of the binary hydrates of methane and carbon dioxide at (**a**) *T* = 273 K and (**b**) *T* = 277 K. Skew crosses = the experimental data [47] of mole fraction carbon dioxide in the hydrate phase; crosses = experimental data [47].

The calculation of hydrate formation pressure as well as the CO_2_ fraction in the hydrate phase in dependence on the gas phase composition were performed for binary CO_2_ + CH_4_ mixtures at temperatures of 273 K and 277 K (Figure 5a,b). The temperatures were chosen in order to describe gas equilibria for both gas–hydrate–ice and gas–hydrate–water.

It is notable that carbon dioxide molecules filled cages better than methane. For example, at the temperature 273 K, the hydrate formation pressure and equimolar composition of the gas phase (50% methane, 50% carbon dioxide), the fraction of CO_2_ molecules in hydrate reached 73% and the fraction of CH_4_ about 27%. At the temperature 277 K, the fractions of gas molecules in hydrate were 70% and 30%, respectively. The ratio of occupancies by CO_2_ and CH_4_ was 2.7:1 at 273 K and 2.3:1 at 277 K. These calculation results agreed well with the experimental data [47]. It has to be noted that with increasing temperature, the fraction of CO_2_ in hydrate decreased. That could be connected with the increase of pressure, which was necessary for gas hydrate formation. In this case, the methane molecules could concur with the carbon dioxide molecules to occupy mainly small hydrate cavities. 

It was connected with the more suitable size of hydrate small cavities for methane molecules in spite of their weaker interactions with surrounding water molecules. In Table 1, the data for binary hydrates formation conditions at several temperatures and gas phase compositions are presented. Calculations were performed for water–gas–hydrate equilibria (*T* = 277 K) and for ice–gas–hydrate equilibria (*T* = 273 K, 258 K).

At relatively low pressures, the solubility of considered gases in ice and water was neglected in our calculations. As could be expected, the equilibrium pressure in systems of gas–hydrate–ice (water) increased with temperature and decreased with a rising amount of carbon dioxide in the gas phase. Analysis of the data also showed nontrivial increasing of the carbon dioxide mole fraction in hydrate with decreasing temperature. That could be connected with the lowering formation pressure while temperature decreased. We could conclude that at low pressure, methane was less favorable than carbon dioxide in cavities occupation.

The formation pressure of binary hydrates rose with increasing methane content in the gas phase and increasing temperature. With temperatures of about 277 K, which corresponds to water temperatures near the bottom of oceans, methane hydrates could form and exist in thermodynamic equilibrium with water and gas at the pressure 4.2 MPa, corresponding to 420 meters in depth (depths of continental slope), whereas for the one-component hydrate of carbon dioxide the formation pressure was lower, 1.2 MPa. At lower temperatures (273 K, 258 K), the decrease of the hydrate formation pressure with the addition of carbon dioxide to methane became not so significant. Thus, with methane content diminishing from 100% to 0%, the change in the formation pressure at *T* = 273 K was about 1.5 MPa and at T = 258 K, it was about 0.8 MPa.

With an increase of methane content in the gas phase, the hydrate formation pressure gradually rose. The formation pressure of double hydrates of methane and carbon dioxide appeared to be a linear function of the methane content in the hydrate. This was remarkable having in mind the significant difference in interaction strengths between the guest molecules (carbon dioxide–carbon dioxide, methane–methane).

The calculations of the formed hydrates composition at different temperatures showed that for replacement of methane in hydrate by carbon dioxide, the low temperature was preferable. 

**Figure 6:**
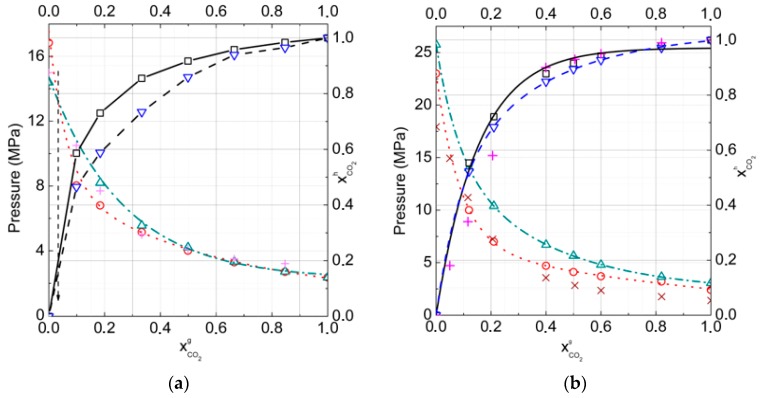
Gas–hydrate–ice (water)phase equilibria for carbon dioxide and nitrogen gas mixtures for cubic structure (CS)-I and CS-II hydrates in comparison with the experimental data, skew crosses [25] and crosses [27], at (**a**) *T* = 272 K and (**b**) *T* = 274 K, respectively ($P_{eq}^{CS-I}$ is dotted by open circles, $P_{eq}^{CS-II}$ is dash-dotted by open triangles, $x_{CO_{2}}^{CS-I}$ is represented by solid open square, and $x_{CO_{2}}^{CS-II}$ is represented by dashed open inverted triangle lines).

To understand the influence of additional nitrogen into the carbon dioxide gas phase on hydrate formation conditions and compositions, the dependencies of the formation pressure and mole fraction of CO_2_ in hydrate on the gas phase composition for temperatures of *T* = 272 K (equilibria of gas–hydrate–ice) and *T* = 274 K (equilibria of gas–hydrate–water), and in the range for CO_2_ mole fractions from 0.0 to 1.0 in the gas phase (Figure 6a,b), were found.

The calculations showed that for all gas phase compositions, the fraction of CO_2_ of more than 0.035 was higher in the hydrate phase relative to the gas phase, and the hydrate structure CS-I appeared to be more stable than structure CS-II. Whereas for all gas phase compositions the fraction of CO_2_ less than 0.035 was higher in the hydrate phase relative to the gas phase, the hydrate structure CS-II appeared to be more stable than structure CS-I. That could be connected with the larger large-to-small cavities ratio in the CS-I structure that was more suitable for CO_2_ molecules. Even at 20% CO_2_ in the gas phase, the CO_2_ fraction in hydrate reached 0.75 at *T* = 272 K and 0.70 at *T* = 274 K. As one can see from Figure 6b, experimental data were described with a reasonable accuracy, at least for relatively low temperatures.

The absence of phase transition CS-I–CS-II in our results at *T* = 274 K could be connected with the roughness of the used approximation, in which we did not take into account the solubility of gases on water. It is not very important at low pressure, but is significant at high pressure, when N_2_ and CO_2_ solubility rapidly increase. On the other hand, one can neglect the solubility of gases on the ice at formation conditions. Another system we considered was CO_2_ + CH_4_ +N_2_ mixtures, which can form hydrates with water or ice. In the gas mixture, the N_2_ mole fraction was fixed at 0.7 and the relative content of CO_2_ and CH_4_ varied from 0.00 CO_2_ to 0.30 CO_2_ in the gas phase. At low concentration of CO_2_ in gas phase pressures of methane replacement by CO_2_ and N_2_ are high and it is not suitable for technology. In this case large cages of hydrate are can be partly filled by nitrogen, but CH_4_ recovery degree would be smaller. 

**Figure 7:**
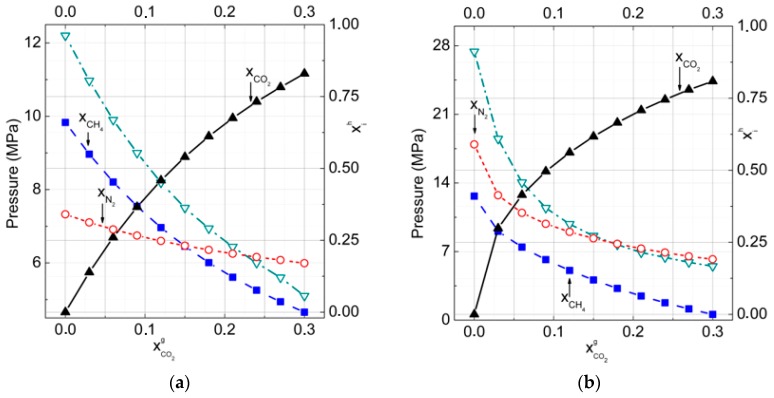
Formation pressure and composition of ternary hydrates carbon dioxide, methane, and nitrogen in dependence on carbon dioxide mole fraction in the gas phase.The nitrogen mole fraction in the gas phase was fixed at a value of 0.7 at temperatures of hydrate formation (**a**) *T* = 273 K, (**b**) *T* = 277 K ($P_{eq}$ is dash-dotted by open inverted triangles,$x_{CH_{4}}$ is dashed by filled squares, $x_{CO_{2}}$ is solid by filled triangles, and $x_{N_{2}}$ is dotted by open circle lines).

In Figure 7a,b, the calculated formation pressures and mole fractions of guests in hydrates of ternary mixtures CO_2_ + CH_4_ + N_2_ are presented. 

The mole fraction of CO_2_ in hydrate increased rapidly with the growth of its content in the gas phase. The fraction of CO_2_ at equilibria both with water and with ice, even at small CO_2_ concentrations, was at least three times higher in the hydrate phase than in the gas mixture (Figure 7a,b). The behavior of N_2_ and CH_4_ guests at equilibria with ice and water were quite different. At equilibrium with ice, CH_4_ occupied many more cavities than N_2_ up to a 0.15 mole fraction of CH_4_ in the gas phase. At higher temperatures, at equilibrium with water and thus for higher formation pressures, the nitrogen became more suitable for occupation of hydrate small cavities.

The solubility of carbon dioxide in water was comparatively high and reached one mole/liter at the hydrate formation pressure and temperature (1.24 MPa at 273 K). At these conditions, the methane solubility was not higher than 0.03 mole/liter. Thus, we could conclude that even at small CO_2_ concentrations in the gas phase, it could be the excess in the reaction mixture that promotes the methane displacement from hydrate.

In Table 2, the calculated *P-T-x* equilibria conditions of gas–hydrate–ice (water) systems at 70% in the gas phase are presented.

If we assume that the formation pressure rose rapidly with the temperature and with lowering CO_2_ content in the gas phase, the obtained data showed very significant increases in the formation pressure after transition from ice to liquid water.

## 4. Conclusions

In this work, the method based on the solid solution theory of clathrate hydrate [34,35,36,37,38] was presented to investigate the effects of the influence of nitrogen on the equilibrium pressure and on the hydrate composition of clathrate hydrates formed from methane + carbon dioxide and nitrogen + carbon dioxide binary gas and methane + carbon dioxide + nitrogen ternary gas mixtures. The comparison of cavities’ filling degrees for binary CO_2_ + CH_4_ with ternary CO_2_ + CH_4_ + N_2_ mixed hydrates showed that carbon dioxide molecules occupied both small and large cavities more preferably than methane, but in the case of ternary mixed hydrate, cavities occupation by N_2_, molecules could be concurred by CO_2_ and CH_4_ molecules.

In all considered systems with and without N_2_, at high and medium CO_2_ content in the gas phase, we found that CO_2_ was more favorable in occupying clathrate hydrate cavities than CH_4_ or N_2_. The addition of N_2_ into the gas phase increased the ratio concentration of CO_2_ in comparison with the concentration of CH_4_ in clathrate hydrates and made gas replacement more effective. The calculation results of the CO_2_ + CH_4_ hydrates confirmed that for all gas phase compositions, the fraction of CO_2_ was higher in the hydrate phase relative to the gas phase, and the hydrate structure CS-I appeared to be more stable than structure CS-II. The mole fraction of CO_2_ in the CO_2_ + CH_4_ + N_2_ gas hydrate increased rapidly with the growth of its content in the gas, and the formation pressure of the CO_2_ + CH_4_ + N_2_ gas hydrate rose in comparison with the formation pressure of the CO_2_ + CH_4_ gas hydrate. Our calculated data were compared with the experimental data [6,25,47], and it was shown that the used theory generally over predicted the experimental data.

## Figures and Tables

**Table 1:** *P-T-x* equilibria of ice (water)–gas–hydrate systems for the CO_2_ + CH_4_ gas mixture.

$x_{CO_{2}}^{g}$ ^a^	$P_{eq}^{258}$^b^ (MPa)	$x_{CO_{2}}^{h}$ ^c^	$P_{eq}^{273}$^d^ (MPa)	$x_{CO_{2}}^{h}$ ^e^	$P_{eq}^{277\;}$^f^ (MPa)	$x_{CO_{2}}^{h}$ ^g^	$S_{t}$ ^h^
1.00	0.55	1.00	1.02	1.00	1.90	1.00	CS-I
0.70	0.69	0.88	1.06	0.86	2.27	0.84	CS-I
0.50	0.83	0.76	1.26	0.73	2.62	0.70	CS-I
0.30	1.03	0.58	1.55	0.54	3.09	0.50	CS-I
0.00	1.66	0.00	2.40	0.00	4.20	0.00	CS-I

^a^$x_{CO_{2}}^{g}$ is the mole fraction of CO_2_ in the gas phase; ^b^ $P_{eq}^{258}$ is the equilibria pressure at *T* = 258 K; ^c^ $x_{CO_{2}}^{h}$ is the mole fraction of CO_2_ in the hydrate phase; ^d^ $P_{eq}^{273}$ is the equilibria pressure at *T* = 273 K; ^e^ $x_{CO_{2}}^{h}$ is the mole fraction of CO_2_ in the hydrate phase; ^f^ $P_{eq}^{277}$ is the equilibria pressure at *T* = 277 K; ^g^ $x_{CO_{2}}^{h}$ is the mole fraction of CO_2_ in the hydrate phase; ^h^ $S_{t}$ is the equilibria type of structure.

**Table 2:** *P*-*T*-*x* equilibria for CO_2_ + CH_4_ + N_2_ +H_2_O in gas–hydrate–ice (water) systems at 70% N_2_ in the gas phase.

$P^{258}$^a^ (MPa)	$P^{265}$^b^ (MPa)	$P^{273\;}$^c^ (MPa)	$P^{274}$^d^ (MPa)	$x_{CO_{2}}^{g}$ ^e^	$x_{CH_{4}}^{g}$ ^f^	$S_{h}^{273}$ ^g^
1.69	1.91	2.76	5.10	0.30	0.00	CS-I
2.08	2.45	3.37	6.44	0.21	0.09	CS-I
2.45	3.00	3.95	7.50	0.15	0.15	CS-I
2.98	3.87	4.74	9.00	0.09	0.21	CS-I
3.97	5.50	6.60	12.20	0.00	0.30	CS-I

^a^$P^{258}$ is the pressure at temperature 258 K; ^b^ $P^{265}$ is the pressure at temperature 265 K; ^c^ $P^{273}$ is the pressure at temperature 273 K; ^d^ $P^{274}$ is the pressure at temperature 274 K; ^e^ $x_{CO_{2}}^{g}$ is the mole fraction of CO_2_ in the gas phase; ^f^ $x_{CH_{4}}^{g}$ is the mole fraction of CH_4_ in the gas phase; ^g^ $S_{h}^{273}$ is a type of hydrate structure.
